# Patient-derived cell lines unveil COL1A2 as a predictor of docetaxel resistance in breast cancer

**DOI:** 10.3389/fonc.2025.1737405

**Published:** 2026-03-02

**Authors:** Yuwei Gao, Rong Wang, Xiaowei Dou, Song Chen, Wenhao Chen, Jinting Liu, Suo Zhu, Jianjun Huang, Qingjun Gao

**Affiliations:** 1 School of Clinical Medicine, Guizhou Medical University, Guiyang, China; 2 Department of Breast Surgery, The Affiliated Hospital of Guizhou Medical University, Guiyang, China; 3 Clinical Research Centre, the Affiliated Hospital of Guizhou Medical University, Guiyang, China; 4 Department of Vascular & Hernia Surgery, The Affiliated Hospital of Guizhou Medical University, Guiyang, China; 5 Department of Breast Surgery, Anshun City People's Hospital, Anshun, China; 6 Department of Thyroid Surgery, The Affiliated Hospital of Guizhou Medical University, Guiyang, China

**Keywords:** breast cancer, HR+/HER2-negative, docetaxel resistance, patient-derived primary cancer cell lines, COL1A2, neoadjuvant chemotherapy, immunohistochemistry, survival analysis

## Abstract

**Introduction:**

Breast cancer is the most common malignant tumor among women worldwide and a major cause of cancer-related mortality. Chemoresistance presents a significant challenge in breast cancer therapy and is a primary driver of tumor recurrence and metastasis. However, effective predictive strategies for chemoresistance remain limited. This study aimed to investigate the mechanisms and predictive models underlying chemotherapy resistance in HR+/HER2− breast cancer.

**Methods:**

Patient-derived primary cancer cell lines (PCCLs) were successfully established from four patients with HR+/HER2− breast cancer (Luminal B, HER2 non-amplified) using primary cell culture techniques. The retention of the original tumors’ pathological characteristics and drug response heterogeneity was confirmed. The sensitivities of PCCLs to taxanes, including docetaxel, were evaluated. RNA sequencing followed by protein–protein interaction (PPI) network analysis was performed to identify candidate genes associated with docetaxel resistance. The expression of COL1A2 was then correlated with pathological complete response (pCR) and recurrence-free survival (RFS) in patients who received neoadjuvant chemotherapy (AC-T regimen). Functional assays were conducted to assess the impact of COL1A2 expression on docetaxel sensitivity in HR+/HER2− breast cancer cells.

**Results:**

PCCLs derived from HR+/HER2− breast cancer exhibited heterogeneous sensitivity to taxanes, including docetaxel. RNA sequencing and PPI network analysis identified COL1A2 as significantly overexpressed in HR+/HER2− breast cancer patients with docetaxel resistance. Elevated COL1A2 expression showed a negative correlation with pCR rates and RFS. In functional assays, higher COL1A2 expression was associated with reduced sensitivity to docetaxel in HR+/HER2− breast cancer cells. These findings were consistent with imaging assessments and postoperative pathological outcomes in patients who underwent neoadjuvant AC-T therapy.

**Discussion:**

Our findings indicate that COL1A2 is associated with reduced chemotherapy sensitivity in HR+/HER2− breast cancer and may serve as a candidate biomarker to guide neoadjuvant taxane selection. This study provides a novel theoretical basis for optimizing neoadjuvant chemotherapy regimens in patients with advanced HR+/HER2− breast cancer.

## 1 Introduction

Breast cancer stands as the most prevalent malignant tumor among women globally. According to statistics released by the International Agency for Research on Cancer (IARC) in 2022, breast cancer accounts for approximately 666,000 deaths annually, representing about 6.9% of all cancer-related fatalities (1). Chemotherapy remains a pivotal treatment modality for breast cancer, particularly for triple-negative and HER2-positive subtypes. Anthracycline- and taxane-based chemotherapy regimens have demonstrated significant efficacy. However, patient responses to chemotherapy vary markedly, and chemoresistance is a critical determinant of chemotherapy failure (2). Neoadjuvant chemotherapy (NACT) holds substantial value in the management of locally advanced breast cancer by reducing tumor burden and enhancing the success rate of breast-conserving surgery (3, 4). Nevertheless, HR+/HER2− breast cancer patients tend to exhibit poor responses to neoadjuvant chemotherapy. Even in highly effective treatment cohorts (such as the Pembrolizumab trial), 70% of patients still fail to achieve pathological complete remission (pCR), thereby limiting the therapeutic impact of NACT in breast cancer (5–8). Consequently, in-depth exploration of the mechanisms underlying breast cancer chemoresistance and the development of effective predictive models is of paramount importance for improving breast cancer treatment outcomes.

In recent years, patient-derived primary cancer cell lines (PCCL) have garnered significant attention due to their ability to accurately predict chemoresistance. PCCL, as *in vitro* culture models directly derived from patient tumor tissues, retain the genetic and microenvironmental characteristics of the tumor, reflecting the *in vivo* features of the primary tumor. This enables precise prediction of patient responses to chemotherapy, making PCCL an ideal tool for investigating tumor resistance mechanisms (9, 10). To date, studies have demonstrated that PCCLs can accurately assess tumor heterogeneity in hepatocellular carcinoma and intrahepatic cholangiocarcinoma, providing references for drug development and personalized treatment targets (11, 12). However, low success rates in PCCL culture and technical limitations have posed barriers to its widespread application. Fortunately, this study overcame these technical hurdles and successfully established four breast cancer PCCL models from HR+/HER2− (Luminal B type, HER2 non-amplified) patients. Validation confirmed that the model preserves the biological characteristics of the original tumor and its sensitivity to first-line standard chemotherapeutic agents, thereby laying a foundation for subsequent research (13).

Type I collagen (COL1), comprising two α1 chains (COL1A1) and one α2 chain (COL1A2), is a crucial member of the collagen family (14). Previous research has predominantly focused on the role of COL1A2 in bone metabolism. Recent studies, however, have highlighted its close association with tumor progression by promoting cancer cell proliferation, invasion, metastasis, and supporting chemoresistance (15). In solid tumors such as gastric cancer and pancreatic cancer, abnormal expression of COL1A2 is closely linked to tumor invasion, metastasis, and chemoresistance (16, 17). Currently, limited research has been conducted on the role of COL1A2 in docetaxel resistance in breast cancer. In this study, we established an HR+/HER2− breast cancer PCCL model and identified COL1A2 as a core gene involved in breast cancer chemoresistance through transcriptome sequencing. Further validation using the TCGA breast cancer dataset and clinical experiments revealed that COL1A2 is highly expressed in taxanes-resistant cells and poorly expressed in sensitive cells. It can participate in chemoresistance formation by regulating tumor cell proliferation. This study innovatively develops a high-success-rate breast cancer PCCL platform and combines gene sequencing with molecular biology techniques to elucidate the mechanisms of COL1A2 in taxanes (docetaxel) resistance in breast cancer. These findings provide a potential target for the precision treatment of HR+/HER2− breast cancer.

## 2 Materials and methods

### 2.1 Patients and tumor samples

All human breast cancer tissue samples were obtained from the Affiliated Hospital of Guizhou Medical University. The prospective collection of diagnostic core (needle) biopsy specimens used for the establishment of patient-derived primary cancer cell lines (PCCLs) was approved by the Ethics Committee of Guizhou Medical University (Approval No. 2019-048), and written informed consent was obtained from all donors. For the exploratory discovery analysis, we restricted the PCCL set to four HR+/HER2-negative lines (BC4, BC5, BC10, BC13) with complete NACT and follow-up information, to minimize inter-subtype heterogeneity and align *in-vitro* phenotypes with clinically annotated sensitivity/non-response. The relevant clinical annotations are reported in **Tables 1**–**3**. Consequently, PCCL RNA-seq served as a hypothesis-generating layer that was cross-validated in an independent postoperative FFPE IHC cohort and public datasets.

**Table 1:** PCCL patient information.

Patient ID	Molecular classification	TNM stage	Clinical stage	NACT
BC4	LuminalB HER2-	T3N1M0	IIIA	AC*4-T*4
BC5	LuminalB HER2-	T2N0M0	IIA	AC*4-T*4
BC10	LuminalB HER2-	T3N1M0	IIIA	AC*6-T*4
BC13	LuminalB HER2-	T2N0M0	IIA	AC*4-T*4

T: Taxanes (including docetaxel, albumin-bound paclitaxel, and paclitaxel).

A: Anthracyclines (including epirubicin, pirarubicin, and doxorubicin).

C: Cyclophosphamide.

(Due to the patient's condition, the treatment cycle for patient BC10 was extended from 8 to 12 cycles.)

**Table 2:** PCCL patient RECIST1.1 standard evaluation.

Patient ID	First diagnosis dimensions (mm)	AC*4		T*4		Miller-Payne grade
		Dimensions (mm)	RECIST score	Dimensions (mm)	RECIST score	
BC4	52 × 48 × 27	40 × 33 × 12	PR	30 × 11 × 20	SD	G3
BC5	15 × 14 × 20	19 × 12 × 15	SD	10 × 8 × 9	PR	G5
BC10	56 × 16 × 45	39 × 18 × 33	SD	32 × 11 × 31	SD	G2
BC13	26 × 14 × 26	19 × 11 × 11	PR	8 × 7 × 8	PR	G5

Target lesion evaluation was conducted according to RECIST 1.1 criteria:

CR (Complete Response): Disappearance of all target lesions.

PR (Partial Response): ≥ 30% decrease in the sum of the longest diameters of target lesions compared to baseline.

PD (Progressive Disease): ≥ 20% increase in the sum of the longest diameters from the smallest recorded value, and/or appearance of new lesions.

SD (Stable Disease): Neither sufficient shrinkage to qualify for PR nor sufficient increase to qualify for PD.

**Table 3:** Expression of molecular markers in breast cancer tissue sections of 4 breast cancer patients.

Patient ID	ER	PR	HER2
BC4	50%	60%	1+
BC5	90%	80%	1+
BC10	60%	60%	1+
BC13	80%	50%	2+

For IHC analysis, we retrospectively retrieved formalin-fixed, paraffin-embedded (FFPE) tumor tissues obtained from surgical resection after treatment at the Affiliated Hospital of Guizhou Medical University. Clinical and imaging data (including RECIST v1.1 assessments of non-pCR cases: stable disease, recurrence or progression) were collected for patients followed for up to 3 years after surgery (18). This retrospective part of the study was approved by the Ethics Committee of the Affiliated Hospital of Guizhou Medical University (Approval No. 2024-489), and, owing to its retrospective nature and the use of de-identified data, the requirement for written informed consent was waived.

#### 2.1.1 Inclusion criteria for tumor patients

Inclusion and exclusion criteria below apply only to the retrospective IHC cohort.

Inclusion criteria

- Patients aged 18–70 years, female.
- Histologically or cytologically confirmed breast cancer, classified as Luminal Type B HER2-negative.
- Tumor size, location, and lymph node status were assessed via imaging (e.g., ultrasound, CT, MRI) or clinical evaluation, making the tumor evaluable.
- All patients had undergone surgery and had a complete and clear Miller-Payne (MP) classification.

Exclusion Criteria

- Patients with severe comorbidities (e.g., heart disease, hepatic and renal dysfunction) or underlying conditions untreatable with drugs.
- Patients unable to tolerate the full cycle of AC-T treatment, such as those with drug allergies, immune system disorders, or severe treatment reactions.
- Patients with active malignant tumors other than breast cancer.
- Other situations precluding trial completion, such as mental disorders or a history of serious adverse drug reactions.

#### 2.1.2 Tumor sample experimental grouping

PCCL Grouping: Four patients with HR+/HER2− breast cancer, undergoing initial full-cycle neoadjuvant chemotherapy, were evaluated using the RECIST criteria. tumor downstaging during NACT was assessed based on clinical imaging data. Patients with stable disease (SD) and progressive disease (PD) were categorized into the resistant group (BC4 and BC10 in this study), while those with partial response (PR) and complete response (CR) were placed in the sensitive group (BC5 and BC13 in this study).

IHC Grouping: Based on clinical imaging data of patients, clinical objective data post-NACT treatment was compared and evaluated using RECIST version 1.1. To mitigate the impact of anthracyclines and cyclophosphamide in combined regimens on tumor downstaging, the evaluation period was restricted to the tumor size after the full cycle of docetaxel drugs, compared to the size after four cycles of anthracyclines and cyclophosphamide without docetaxel. The docetaxel-resistant group was defined as a reduction of < 30% and an increase of < 20% in the sum of the longest diameters of target lesions, indicating stable disease (SD). The sensitive group was defined as a reduction of ≥ 30% in the sum of the longest diameters of target lesions, indicating partial remission (PR).

### 2.2 Establishment of patient-derived primary breast cancer cell lines

Breast tumor tissues (BC4, BC5, BC10, and BC13) were obtained from diagnostic biopsy specimens of four patients with HR+/HER2− breast cancer (Luminal B type, HER2 non-amplified). The tissues were placed in D-Hank’s balanced salt solution containing 500 U/mL penicillin and 500 μg/mL streptomycin, transported to the laboratory at low temperature, and stored at 4 °C. After washing the specimens twice with sterile D-Hank’s balanced salt solution (about 3 mL each time), the tissues were minced and added to D-Hank’s balanced salt solution containing 140 U/mL type II collagenase (17101015, Thermo Fisher Scientific, USA). Digestion was performed on a shaker at 37°C and 100 rpm for approximately 1 h. Subsequently, the cells were centrifuged at 1000 rpm for 10 min, the supernatant was discarded, and the cell suspension was cultured in BCMI medium (SFCtech, Beijing, China) at 37°C and 5% CO_2_. After cell proliferation, the medium was changed to high-glucose DMEM medium. After at least two trypsin-EDTA/TrypLE digestions and mild mechanical dissociation to remove stromal cells, the expression of ERα, PR, and HER2 in PCCL (BC4, BC5, BC10, BC13) and their corresponding breast cancer tissues was verified by staining. The results demonstrated that the expression of molecular markers in primary breast cancer cells was consistent with that in the corresponding tissues.

### 2.3 Chemosensitivity screens

To predict the sensitivity of breast cancer cells to docetaxel, represented by docetaxel, the proliferation ability of four PCCL cells was assessed using the CCK-8 method. Breast cancer cells were seeded in 96-well plates and cultured for 24 hours. Different doses of docetaxel (5000 nM, 1250 nM, 312 nM, 78 nM, 19.5 nM, 4.88 nM, 1.22 nM, and 0 nM) were used for culture. Each concentration was replicated three times and cultured for 72 hours. Cytotoxicity was determined using a CCK-8 kit, with absorbance measured at 450 nm after 1 hour of incubation. The average cell viability at each drug concentration was determined by normalization with the negative control value, and the proliferation curve was plotted to determine the half-inhibitory concentration (IC_50_) value.

### 2.4 Gene expression bioinformatic analysis

#### 2.4.1 Screening of significantly differentially expressed genes in docetaxel-resistant and sensitive strains of PCCL

Total RNA was extracted from patient-derived primary cancer cell lines (PCCLs) and quantified with Qubit; integrity was assessed on an Agilent 2100 Bioanalyzer (RNA Nano 6000 assay; RIN ≥ 7). Poly(A)+ mRNA was purified using oligo(dT) magnetic beads and fragmented with divalent cations at elevated temperature. Strand-specific (dUTP) libraries were constructed according to the manufacturer’s instructions (NEB, USA): first-strand synthesis with random hexamers/M-MuLV reverse transcriptase, second-strand synthesis with DNA polymerase I and RNase H (dUTP incorporation), 3′-adenylation, adaptor ligation, AMPure XP size selection yielding ~370–420 bp inserts, and PCR with a high-fidelity polymerase, followed by Bioanalyzer QC. Indexed libraries were clustered on a cBot (TruSeq PE Cluster Kit v3-cBot-HS) and sequenced on an Illumina NovaSeq 6000 to generate paired-end 150-bp reads.

Raw FASTQ files underwent adapter/quality trimming with fastp v0.19.7 (reporting Q20/Q30 and GC-content metrics; typical parameters: -g -q 5 -u 50 -n 15 -l 150). Clean reads were aligned to the human reference genome (hg38) using HISAT2 v2.0.5 (splice-aware; indices built from the gene model), and gene-level counts were obtained with featureCounts v1.5.0-p3. For downstream analyses, expression values were normalized using standard approaches (TMM for count-based modelling and TPM/FPKM where appropriate for display). Per-sample QC metrics are summarized in **Supplementary Table S1**.

To identify transcripts associated with docetaxel response, docetaxel-resistant PCCLs (BC4, BC10) were compared with docetaxel-sensitive controls (BC5, BC13). Genes unexpressed or lowly expressed across all samples (no sample with CPM > 1) were excluded prior to modelling. Differential expression was performed with edgeR, defining DEGs at Benjamini–Hochberg FDR < 0.05 and |log2FC| > 1 (pre-filter CPM > 1). Results were visualized with volcano plots and functionally annotated by Gene Ontology/KEGG terms using clusterProfiler. All analyses were conducted in R 4.4.2.

**Figure 1:**
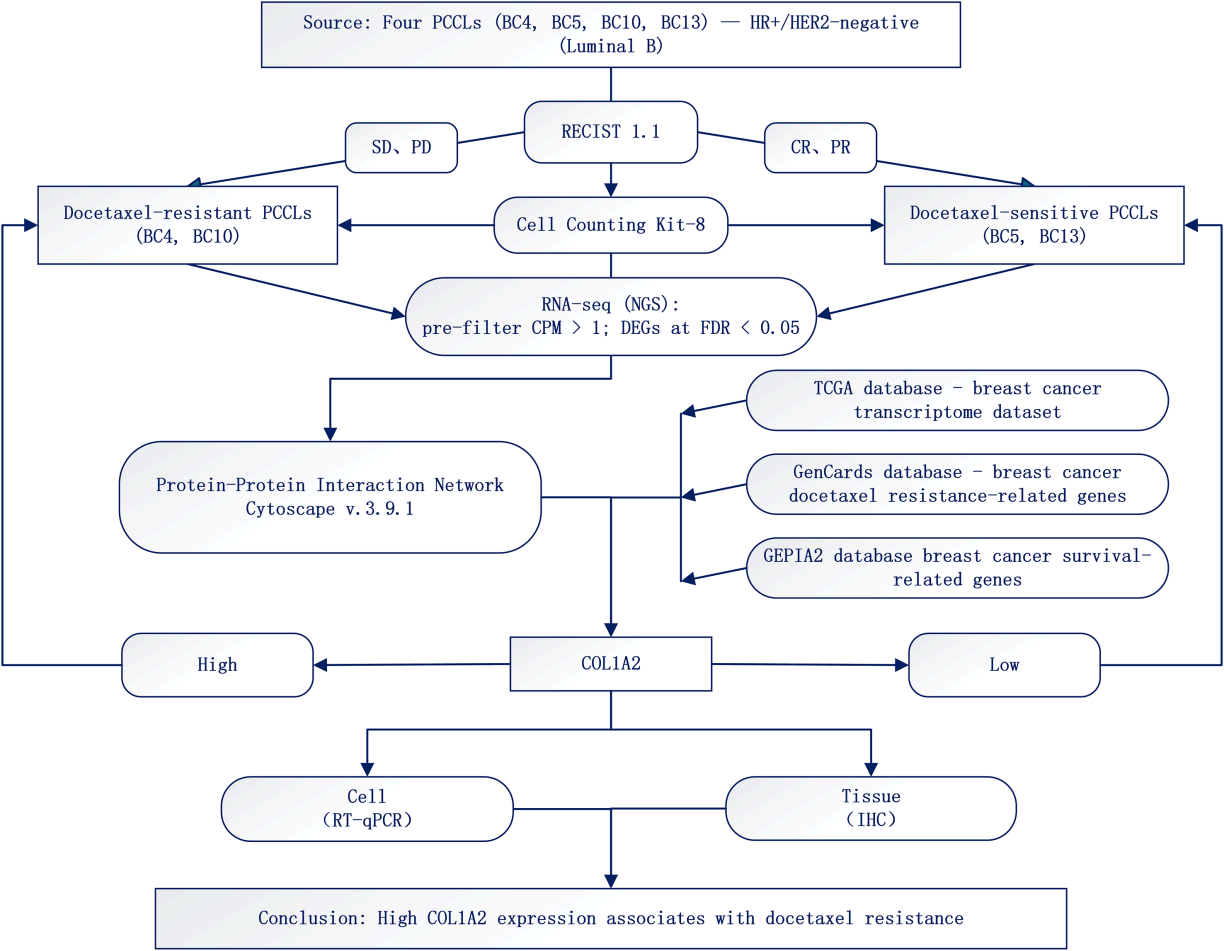
Flowchart.

Protein–protein interaction networks were generated from STRING (confidence ≥ 0.70) and visualized in Cytoscape v3.9.1. Key regulatory nodes were ranked by changes in gene/protein activity and network topology (degree/betweenness), and the top 10 hub genes were screened. Cross-dataset interrogation using GEPIA, TIMER, UALCAN, Kaplan–Meier survival analysis and ROC Plotter-Online indicated that COL1A2 is highly expressed in TCGA tumors—particularly breast cancer—and is associated with poorer recurrence-free survival and reduced docetaxel sensitivity (**Figure 1**).

#### 2.4.2 Integration of public data resources

##### 2.4.2.1 Data source

TCGA-BRCA STAR-counts data and corresponding clinical information were extracted from The Cancer Genome Atlas (TCGA) database. The TCGA-BRCA data were divided into two groups: Normal and tumor (113 normal samples and 1,118 tumor samples). Seven hundred genes related to taxane resistance in breast cancer (relevance score > 100) were downloaded and screened from the GeneCards database. The top 500 genes related to breast cancer survival were obtained from GEPIA2. Venn diagrams were drawn using Venny2.1.0.

##### 2.4.2.2 Identification of differentially expressed genes in TCGA database

The limma, edgeR, pheatmap, and ggplot2 packages in R (4.4.2) software were utilized to process the breast cancer transcriptome dataset in the TCGA database. Differentially expressed genes between normal breast tissue and breast cancer tissue were analyzed under the conditions |log2FC| > 1 and P < 0.05, and visualized using volcano plots and heatmaps.

##### 2.4.2.3 Survival analysis of breast cancer patients

The median expression of the target gene in breast tissue was used as the cutoff point to divide patients into high- and low-expression groups. Kaplan-Meier survival curves for the high and low expression groups were plotted using the "survival" package in R software (4.4.2), and differences were compared. A P-value <0.05 indicated statistical significance.

##### 2.4.2.4 Drug sensitivity analysis

The "oncoPredict" package was employed to predict the half-inhibitory concentration (IC_50_) value. The sensitivity of genes to docetaxel was analyzed using GDSC drug-response data and gene expression data.

#### 2.4.3 Obtaining target genes

The differentially co-expressed genes from the TCGA database’s breast cancer transcriptome dataset, the top 500 BRCA survival-related genes from the GEPIA2 database, genes related to taxane (docetaxel) resistance in breast cancer from the GeneCards database, and significantly differentially expressed genes from docetaxel-resistant and sensitive PCCL strains were integrated to construct a Venn diagram, thereby identifying the target genes.

### 2.5 qRT-PCR

Quantitative real-time PCR (qRT-PCR). Total RNA was extracted from cells at ~70–75% confluence using TRIzol (Thermo Fisher Scientific, USA; Cat# TSP413), quantified on NanoDrop, and reverse-transcribed with the SynScript®III RT SuperMix (Thermo Fisher Scientific; Cat# TSK314S) following the manufacturer’s protocol. qRT-PCR was performed on a QuantStudio StepOne Plus (Applied Biosystems) using ArtiCan^CEO^ SYBR qPCR Mix (Cat# TSE401). Relative expression was calculated by the 2^^-ΔΔCt^ method with ACTB as the endogenous control. Primer sequences are listed in **Table 4**.

**Table 4:** Primer sequences.

Primer name	Primer sequence (5' to 3')
1-COL1A2-F	TCTCTCAGACCCAAGGACTATGA
1-COL1A2-R	TTCGCCAGTAGAGAAATCACAGT
β-actin-F	CCTTCCTGGGCATGGAGTC
β-actin-R	TGATCTTCATTGTGCTGGGTG

### 2.6 Immunohistochemistry

Immunohistochemical analysis was performed on tumor tissue samples from 54 patients with luminal B-type HER2-negative breast cancer. After fixation with 4% paraformaldehyde, dewaxing, and hydration, antigen retrieval was carried out using citrate buffer. Endogenous peroxidase activity was blocked with 3% hydrogen peroxide, and nonspecific binding sites were blocked with 5% normal goat serum. Slides were incubated with primary antibodies against Ki67, E-cadherin, N-cadherin, and COL1A2 at 4°C overnight, washed with TBST, incubated with HRP-labelled secondary antibodies, and developed with DAB. Haematoxylin was employed for counterstaining. Two pathologists evaluated the immunostaining results in a blinded fashion. Positive signals were predominantly localized in the extracellular matrix. Five high-power fields were randomly selected, and the positive rate of 200 tumor cells was determined. Scoring was based on staining intensity (0–3 points) and the percentage of positive cells (0–4 points), with the product result determining the positive grade: 0 points for grade 0 (negative), 1–2 points for grade 1, 3–4 points for grade 2, and 4 or more points for grade 3 (positive).

### 2.7 Statistical analyses

Continuous variables were summarized as mean ± SD for normally distributed data or median (IQR) otherwise. Normality was assessed with the Shapiro–Wilk test and homoscedasticity with Levene’s test. Between-group comparisons used Student’s t test; when assumptions were violated, the Mann–Whitney U test was applied. For repeated measurements on the same samples (e.g., pre–post or paired designs), paired t tests (or Wilcoxon signed-rank tests for non-normal data) were used. IC_50_ values were obtained by fitting four-parameter logistic (4PL) dose–response curves using GraphPad Prism 10 (nonlinear least squares), and differences in IC_50_ between docetaxel-resistant and -sensitive groups were compared with the appropriate (unpaired) t test or its nonparametric alternative. Categorical variables were presented as n (%), and group differences were evaluated with the χ² test or Fisher’s exact test as appropriate. Associations between clinicopathological characteristics and gene mutation/status and analyses of clinical predictors of the primary outcome were performed in SPSS 27.0 using logistic regression; for RECIST analyses, SD/PD was coded as 1 and CR/PR as 0; results are reported as odds ratios (ORs) with 95% confidence intervals (CIs). Linear correlations were assessed with the Pearson correlation coefficient (or Spearman when distributions were non-normal). Where applicable, multiple testing was controlled using the Benjamini–Hochberg false discovery rate (FDR) procedure. All tests were two-sided, with P < 0.05 considered statistically significant. Unless otherwise specified (e.g., visualization in volcano plots), inference was based on FDR-adjusted P-values.

## 3 Results

### 3.1 Subject demographic and treatment characteristics

The breast cancer diagnostic biopsy samples included in the study were from patients with invasive ductal carcinoma, which was histologically confirmed as luminal B type, HER2 non-amplified, and clinically staged II-III. All patients received at least 8 cycles of AC-T neoadjuvant chemotherapy (**Table 4**). To exclude the potential impact of anthracyclines and cyclophosphamide on the clinical evaluation of docetaxel, imaging data were collected at three stages: initial admission, after anthracycline and cyclophosphamide treatment, and before surgery (i.e., after completing docetaxel treatment). RECIST version 1.1 was used to evaluate tumor downstaging. The results showed that imaging of patients BC4 and BC13 demonstrated significant tumor shrinkage after anthracycline and cyclophosphamide treatment, with the sum of the longest diameters of the target lesions decreasing by approximately 33.0% and 37.9%, respectively. These were evaluated as PR. In the subsequent neoadjuvant treatment, the tumor size of patient BC4 tended to stabilize with docetaxel treatment and was evaluated as SD, while patient BC13 remained highly sensitive to docetaxel. During docetaxel treatment, tumor changes in patients BC5 and BC13 were significantly different. Patient BC5 was resistant to anthracycline and cyclophosphamide, with the sum of the longest diameter of the tumor decreasing by only 0.06%. However, under docetaxel treatment, the tumor shrank by about 41.3%, showing high sensitivity. In contrast, patient BC10 responded flatly to both treatments, with the tumor size decreasing by only 17.8% during docetaxel treatment. According to the Miller-Payne grading system for evaluating the effect of neoadjuvant chemotherapy, patients BC5 and BC13 reached G5, patient BC4 was G3, and patient BC10 was G2.

### 3.2 Characterization and validation of PCCLs

**Figure 2:**
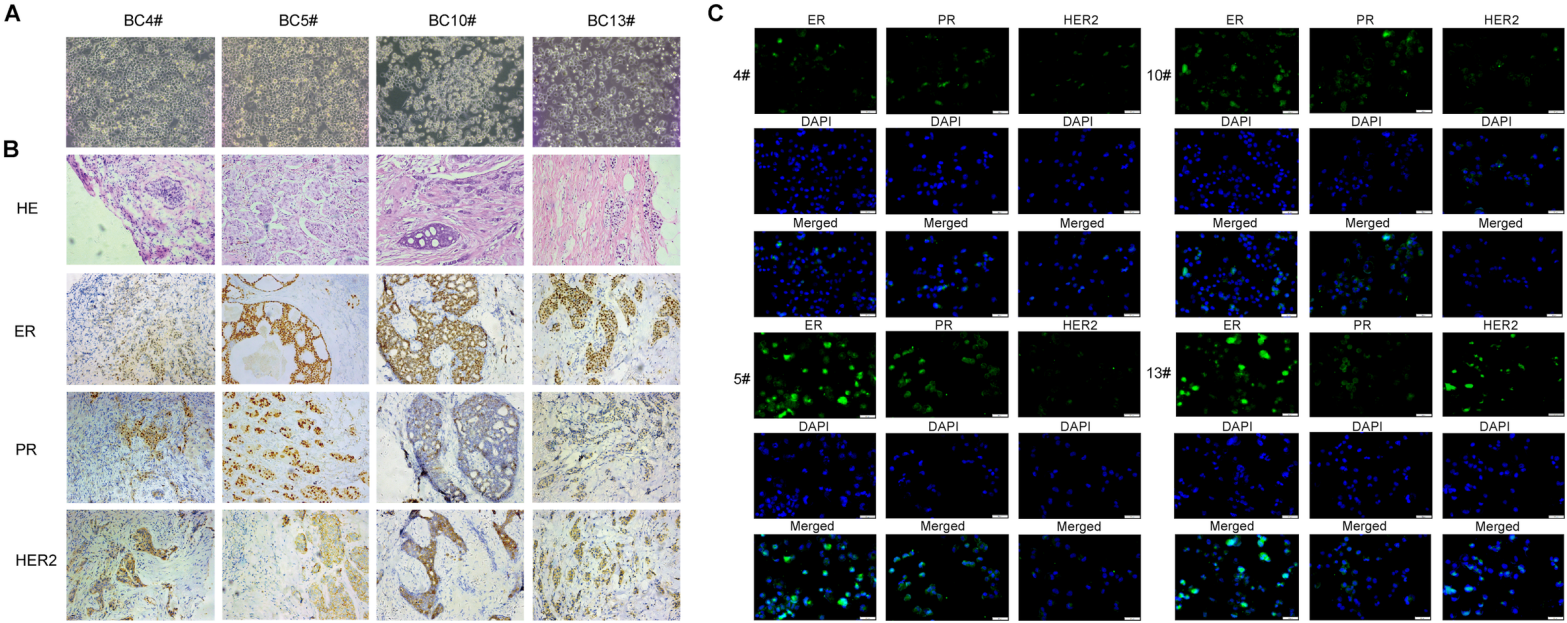
IHC and immunofluorescence staining in primary breast cancer cells. **(A)** Growth morphology of primary cancer cell lines (PCCLs) BC4, BC5, BC10, and BC13 under drug-free conditions. **(B)** IHC staining (HE, ER, PR, HER2) of tissue sections from BC4, BC5, BC10, and BC13. **(C)** Immunofluorescence staining for ER, PR, and HER2 in primary cells BC4, BC5, BC10, and BC13.

After collecting breast cancer tissue from breast surgery, we successfully established 4 primary breast cancer cell lines that could be passaged multiple times. It was observed that the BC4, BC5, and BC13 cell lines proliferated faster, and the cells were dispersed, whereas the BC10 cell line proliferated more slowly, and the cells were tightly bound (**Figure 2A**). To identify the histological consistency between primary cell lines and primary breast cancer tumor tissues, we performed IHC and immunofluorescence staining of ERα, PR, and HER2 in the four primary cell lines and their corresponding breast cancer tissues. The results showed that the expression of these molecular markers in the primary cell lines was consistent with that in the corresponding breast cancer tissues (**Figure 2**).

### 3.3 Chemosensitivity profiling to docetaxel

**Figure 3:**
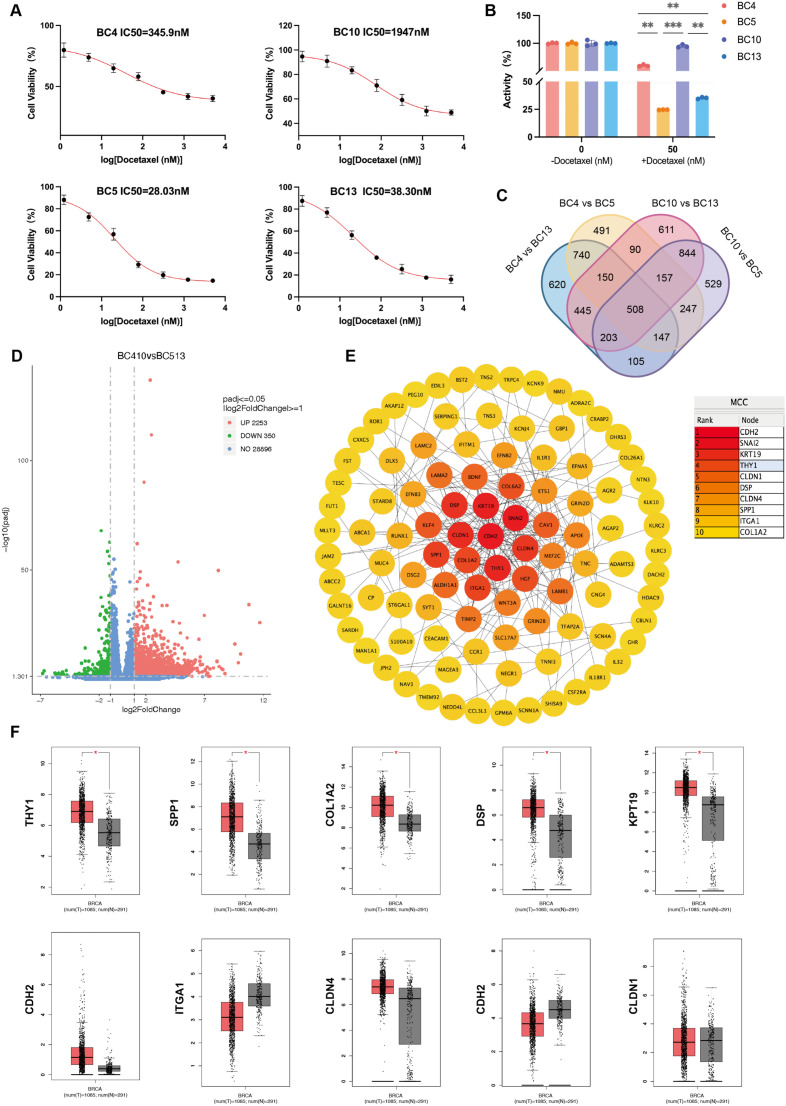
Screening of target genes related to docetaxel sensitivity. **(A)** IC_50_ values of docetaxel in primary breast cancer cells (BC4, BC5, BC10, and BC13) measured by CCK-8 assay. **(B)** Growth comparison of cells treated with 0 nM vs 50 nM docetaxel. **(C)** Venn diagram of differentially co-expressed genes between docetaxel-sensitive and -resistant lines (CPM > 1, FDR < 5%). **(D)** Volcano plot of differentially expressed genes (|log2FC| > 1, P < 0.05). **(E)** PPI network constructed via STRING, with top 10 hub genes identified using Cytoscape v3.9.1. **(F)** Expression analysis of five upregulated genes (DSP, KRT19, SPP1, THY1, COL1A2) in breast cancer based on GEPIA. *P < 0.05, **P < 0.01, ***P < 0.001.

To predict the chemosensitivity of breast cancer cells to docetaxel, we used the CCK-8 assay to detect 4 primary breast cancer cell lines (**Figure 3A**).Under the same concentration gradient of docetaxel treatment, the average IC_50_ value of BC10 was significantly higher than those of the other cell lines, while the average IC_50_ value of BC5 was the lowest. This indicates that BC10 is relatively insensitive to docetaxel treatment, while BC5 shows higher sensitivity (**Figure 3B**). It is worth noting that BC10 was still assessed as G2 by the Miller-Payne classification after extended treatment cycles. In addition, BC5 was resistant to anthracyclines and cyclophosphamide in neoadjuvant chemotherapy but showed high sensitivity to docetaxel, which is consistent with the results of *in vitro* experiments.

### 3.4 Gene expression analysis in PCCLs

To explore the resistance mechanism of docetaxel, we performed whole genome RNA sequencing on primary breast cancer cell lines from the docetaxel-resistant group (BC4, BC10) and the sensitive group (BC5, BC13) to analyze gene expression differences and investigate the resistance mechanism of docetaxel. After differential expression analysis of RNA sequencing data, 2603 genes were found to be significantly differentially expressed between the resistant and sensitive groups (|log2FC| > 1, P < 0.05), with up-regulated genes marked in red and down-regulated genes marked in green (**Figure 3D**).The Venn diagram showed that there were 508 differentially co-expressed genes between the resistant and sensitive groups (**Figure 3C**).

In addition, we constructed a protein-protein interaction (PPI) network using the STRING online database and imported it into Cytoscape v.3.9.1 for analysis. Based on the changes in protein/gene activity and their regulatory mechanisms, we identified 10 key regulatory factors: CDH2, SNAI2, KRT19, THY1, CLDN1, DSP, CLDN4, SPP1, ITGA1, and COL1A2 (**Figure 3E**).The GEPIA database was further used to analyze the differential expression of the above genes in breast cancer. The results showed that COL1A2, DSP, KRT19, SPP1, and THY1 were upregulated in breast cancer, and the expression differences were statistically significant (**Figure 3F**).

### 3.5 TCGA database differentially expressed genes combined with PCCL model to screen target genes

**Figure 4:**
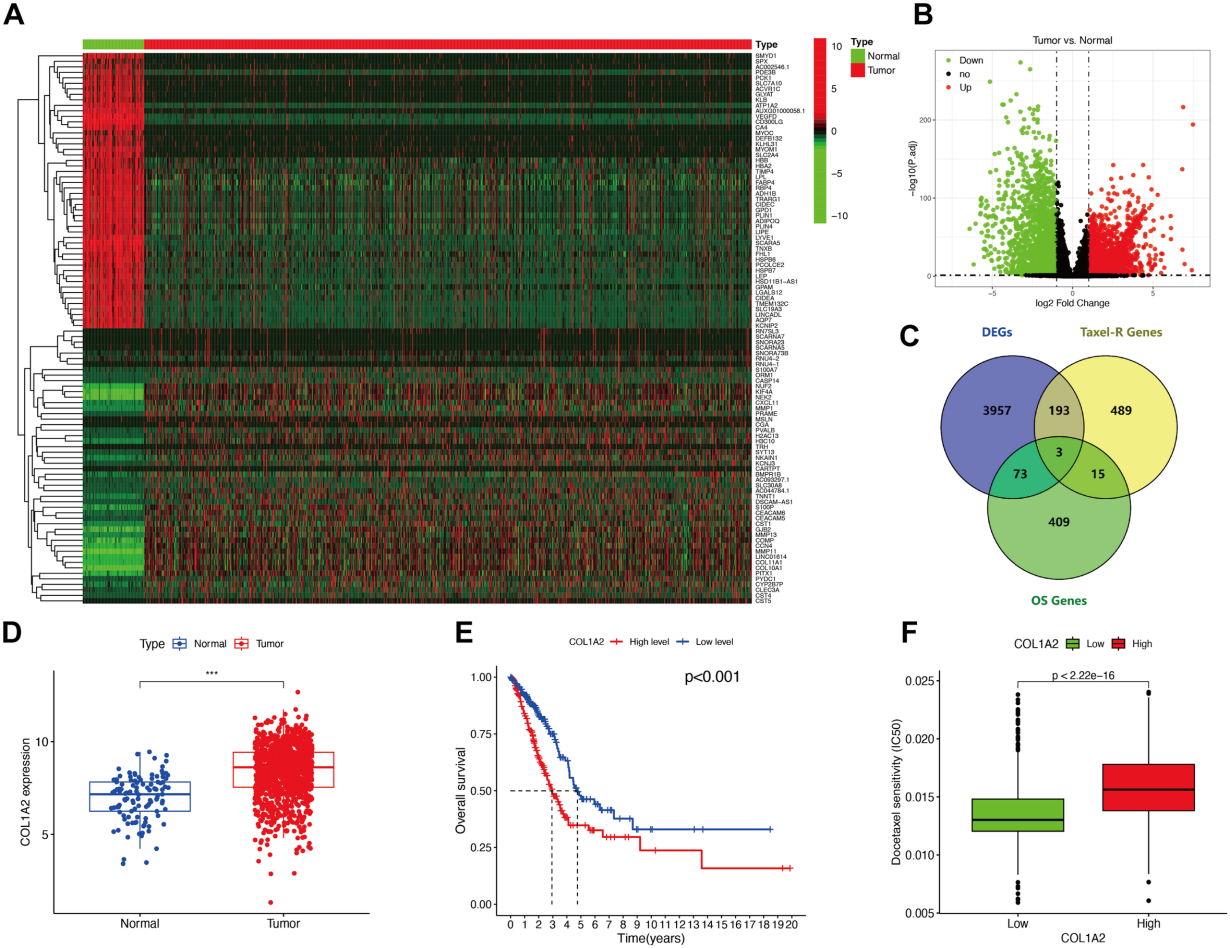
Validation of COL1A2 as a potential target gene. **(A)** Transcriptomic analysis of breast cancer datasets from TCGA. **(B)** Volcano plot identifying 4226 differentially expressed genes (|log2FC| > 1, P < 0.05). **(C)** Venn diagram of overlapping genes across GEPIA2, GeneCards, and TCGA-BRCA datasets. **(D)** COL1A2 is significantly upregulated in breast cancer tissues (P < 0.001). **(E)** High COL1A2 expression correlates with shorter median survival (P < 0.001). **(F)** Docetaxel IC50 is significantly higher in cells with high COL1A2 expression (P < 1×10^-15^). ***P < 0.001.

The breast cancer transcriptome dataset from the TCGA database was exported, and the TCGA-BRCA data were divided into normal and tumor groups. Differential genes between normal breast tissue and breast cancer tissue were analyzed. The conditions were |log2FC| > 1, P < 0.05, and 4226 differentially co-expressed genes were identified (**Figures 4A, B**). The first 500 breast cancer survival-related genes obtained from GEPIA2, the 700 taxane (docetaxel) resistance-related genes obtained from GeneCards, and the TCGA-BRCA differentially co-expressed genes were jointly represented in a Venn diagram (**Figure 4C**). The intersecting genes were identified: PMM2, COL1A2, KRT5. After comparing the 10 significantly differentially co-expressed genes (CDH2, SNAI2, KRT19, THY1, CLDN1, DSP, CLDN4, SPP1, ITGA1, and COL1A2) screened from PCCL cell lines, we found that COL1A2 was a differentially co-expressed gene that simultaneously satisfied the above data sets.

COL1A2 was validated and analyzed (**Figures 4D–F**), and we found that COL1A2 expression was significantly upregulated in breast cancer (P < 0.001). Kaplan-Meier survival curves of COL1A2 high and low expression groups were plotted. It was observed that overall survival (OS) was significantly lower in the high expression group compared to the low expression group (P < 0.001). The median value was used for estimation, and the median survival of patients with high COL1A2 expression was nearly 2 years shorter than that of patients with low COL1A2 expression. GDSC drug sensitivity data were used to analyze the sensitivity of COL1A2 to docetaxel. We found that when COL1A2 was highly expressed, the IC_50_ of docetaxel was significantly higher than when COL1A2 was lowly expressed (P < 1×10^-15^), indicating that high expression of COL1A2 can induce cells to have stronger resistance to docetaxel. Therefore, we believe that the expression of COL1A2 may serve as a potential biomarker to predict the response of tumor patients to docetaxel chemotherapy, and high expression of COL1A2 may indicate poor docetaxel treatment efficacy in patients.

### 3.6 qRT-PCR validation of COL1A2

To confirm the differential expression of COL1A2 in docetaxel-resistant and -sensitive cell lines, we measured the expression level of COL1A2 mRNA in BC4 and BC10 cell lines, which are resistant to docetaxel (DTX), at standardized doses using qRT-PCR analysis. The results showed that the expression of COL1A2 in resistant cell lines (BC4, BC10) was significantly higher than in sensitive cell lines (BC5, BC13) (**Figure 4D**).

### 3.7 Immunohistochemical evaluation of the correlation between COL1A2 protein expression and drug resistance

To study the effect of COL1A2 on drug resistance in patients with luminal B HER2-negative breast cancer receiving neoadjuvant chemotherapy (NACT), we performed immunohistochemistry (IHC) to detect the expression level of COL1A2 in primary tumor tissues from 54 patients treated with the AC-T regimen. The results (**Table 5**) showed that among the 22 patients with Miller-Payne grades G1 and G2, 54.55% (12/22) had increased COL1A2 expression; while among G3 patients, 40.00% (6/15) had increased COL1A2 expression, and only 11.76% (2/17) of patients with G4/G5 had increased COL1A2 expression. Additionally, increased COL1A2 expression was mainly found in the cytoplasm and extracellular matrix of cancer tissues. It is worth noting that, in patients with Miller-Payne grades G1 and G2, the neoadjuvant chemotherapy (NACT) treatment effect was better in areas with low COL1A2 expression. These findings suggest that COL1A2 expression may be associated with the response and resistance of breast cancer patients to neoadjuvant chemotherapy, providing an important experimental basis for subsequent studies.

**Table 5:** Correlation between clinicopathological characteristics and COL1A2 expression.

Characteristics	High	Low	Total number (%)	χ²	P-value
Age (years)				0.713	0.399
< 50	10	21	31 (57.40)		
≥ 50	10	13	23 (42.60)		
Menopausal status				3.469	0.063
Pre	9	24	33 (61.10)		
Post	11	10	21 (38.90)		
Ki67 value				0.091	0.763
< 14%	4	8	12 (22.22)		
≥ 14%	16	26	42 (77.78)		
pT stage				20.207	< 0.001
T1	1	5	6 (11.11)		
T2	5	24	29 (53.70)		
T3	4	4	8 (14.81)		
T4	10	1	11 (20.37)		
pN stage				0.678	0.712
N0	9	19	28 (51.85)		
N1	6	9	14 (25.93)		
N2+N3	5	6	11 (20.37)		
AJCC clinical classification				11.268	0.004
Stage I	1	4	5 (9.26)		
Stage II	5	22	27 (50.00)		
Stage III+IV	14	8	22 (40.74)		
Pathological status				6.323	0.012
pCR	1	12	13 (24.07)		
non-pCR	19	22	41 (75.93)		
MP				7.605	0.022
G1+G2	12	10	22 (40.74)		
G3	6	9	15 (27.78)		
G4+G5	2	15	17 (31.48)		
RFS				9.535	0.002
Recurrence-Free	11	31	42 (77.78)		
Recurrence	9	3	12 (22.22)		

**Figure 5:**
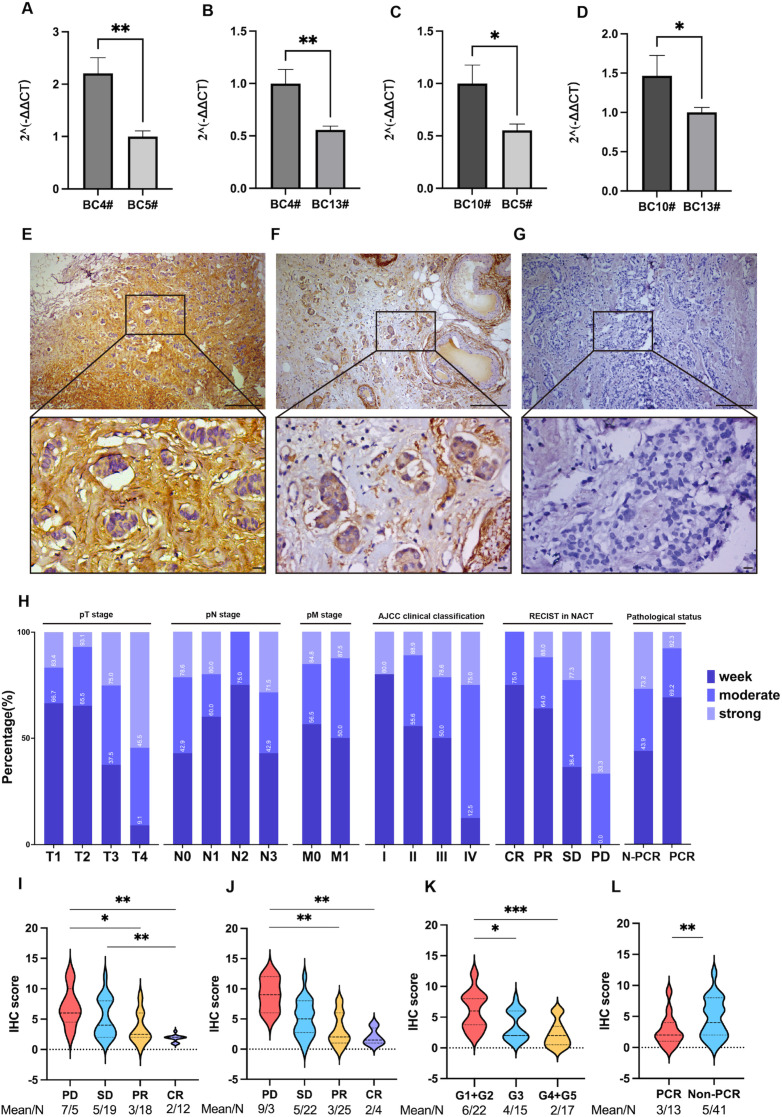
Expression pattern and clinical correlation of COL1A2 in breast cancer. **(A, B)** COL1A2 expression is significantly higher in docetaxel-resistant cell line BC4 compared to sensitive cell lines BC5 and BC13 (P < 0.01). **(C, D)** Similar significant differences observed between resistant cell line BC10 and sensitive lines BC5 and BC13 (P < 0.05). **(E–G)** Representative IHC images showing high, medium, and low COL1A2 expression in breast cancer tissues (10× and 40× magnification). **(H)** Stacked percentage bar charts showing the distribution of TNM stage, AJCC grade, NACT response, and postoperative pathology across different COL1A2 expression groups. **(I–L)** IHC scoring system comparisons: docetaxel response during NACT, overall treatment efficacy, Miller–Payne grading, and pCR evaluation system. *P < 0.05, **P < 0.01, ***P < 0.001.

Interestingly, we observed the IHC experimental results and found that in areas with high COL1A2 expression, the extracellular matrix of tumor cells showed strong staining. Tumor cells surrounded by type I collagen deposition responded less to chemotherapy, whereas in areas with low COL1A2 expression, the staining was more uniform, and the tumor cells responded better to chemotherapy (**Figures 5E–G**).

### 3.8 The clinical correlation analyses of COL1A2 expression and prognosis in breast cancer patients

We collected 54 clinical tissue samples, all of which had complete treatment follow-up data and gene expression data. The patient characteristics were as follows: 31 patients (57.40%) were under 50 years old at diagnosis, more than 60% of the patients (33 patients) were premenopausal at diagnosis, and 11 patients (20.37%) were locally advanced at diagnosis. Lymph node staging: N0 (28 patients, 51.85%), N1 (14 patients, 25.93%), N2/N3 (11 patients, 20.37%). Clinical staging: stage I (5 patients, 9.26%), stage II (27 patients, 50.00%), stage III/IV (22 patients, 40.74%). After receiving neoadjuvant chemotherapy, the ratio of patients who achieved pathological complete remission (pCR) to those who did not achieve pCR was approximately 1:3. The 3-year follow-up showed that the ratio of disease-free recurrence to disease-related recurrence (including local recurrence and distant metastasis) was approximately 3:1.

### 3.9 High COL1A2 is associated with docetaxel resistance in breast cancer cells.

To further evaluate the clinical significance of COL1A2 in docetaxel chemotherapy resistance in breast cancer, we divided patients into two stages according to the imaging B-ultrasound results at different stages of NACT treatment and the RECIST guidelines: anthracycline combined with cyclophosphamide treatment stage and docetaxel treatment stage. According to the RECIST criteria, the treatment effect was divided into a sensitive group (CR, PR) and a resistant group (SD, PD), and the relationship between COL1A2 expression and clinical efficacy during neoadjuvant therapy in patients with primary breast cancer was evaluated (**Table 6**). The results showed that during docetaxel treatment, COL1A2 was highly expressed in 17 patients (85%) in the resistant group, while only 3 patients (15%) in the sensitive group were lowly expressed. Moreover, the expression level of COL1A2 was significantly negatively correlated with the remission rate of docetaxel treatment (χ²=11.153, P = 0.001), while during the treatment of anthracycline combined with cyclophosphamide, the expression level of COL1A2 was not significantly correlated with its remission rate (χ²=0.374, P = 0.572). The IHC score was converted into a violin plot to evaluate the efficacy of docetaxel treatment during NACT, the total efficacy of treatment, the Miller-Payne grading evaluation of pathology after neoadjuvant treatment, and the pCR assessment. It was found that the median COL1A2 score decreased in order according to PD~CR; G1~G5; non-pCR~pCR status (**Figures 5I–L**).

**Table 6:** Expression of COL1A2 in patients with primary breast cancer undergoing chemotherapy and evaluation of clinical efficacy during neoadjuvant therapy.

Characteristics	High	Low	Total number (%)	χ²	P-value
Anthracycline RECIST				0.374	0.572
CR-PR	6	13	19 (35.19)		
SD-PD	14	21	35 (64.81)		
Docetaxel RECIST				11.153	0.001
CR-PR	3	21	24 (44.44)		
SD-PD	17	13	30 (55.56)		
Total RECIST				2.719	0.257
CR-PR	12	16	28 (51.85)		
SD-PD	6	17	23 (42.59)		

Anthracycline RECIST: Anthracycline response per RECIST 1.1.

Docetaxel RECIST: Docetaxel response per RECIST 1.1.

Univariate logistic regression analysis showed that in HR+/HER2− breast cancer patients, high COL1A2 expression levels were significantly associated with age (OR = 3.6; 95%CI: 1.129-11.478; P = 0.03), menopausal status (OR = 7.0; 95%CI: 1.993-24.581; P = 0.002), tumor size (T4 vs The expression level of COL1A2 was significantly correlated with the remission rate during docetaxel treatment (OR = 8.608; 95%CI: 1.325-55.928; P = 0.024). In addition, high expression of COL1A2 was significantly associated with poor response rate (OR = 7.356; 95%CI: 1.055-51.316; P = 0.044) and recurrence-free survival rate of docetaxel (DTX) chemotherapy (**Table 7**).

**Table 7:** Univariate and multivariate Logistic regression analysis of COL1A2 expression and clinical pathological characteristics in patients with primary breast cancer.

Covariates	Univariate analysis		Multivariate analysis	
	OR (95% CI)	P-value	OR (95% CI)	P-value
Age (< 50 versus ≥ 50 years)	3.600 (1.129-11.478)	0.03	0.521 (0.043-6.391)	0.611
Menopausal status(Pre versus Post)	7.000 (1.993-24.581)	0.002	7.365 (0.571-94.916)	0.126
Ki67 value (< 14% versus ≥ 14%)	1.231 (0.318-4.758)	0.763		
pT stage				
T2 versus T1	1.042 (0.099-10.959)	0.973		
T3 versus T1	5.000 (0.388-64.387)	0.217		
T4 versus T1	50.000 (2.559-976.970)	0.01		
pN stage				
N1 versus N0	1.407 (0.383-5.176)	0.607		
N2+N3versus N0	1.759 (0.422-7.333)	0.438		
AJCC clinical classification				
Stage II versus stage I	0.909 (0.83-9.989)	0.938		
Stage III+IV versus stage I	7.000 (0.663-73.929)	0.106		
Anthracycline RECIST (SD-PD versus CR-PR)	1.444 (0.444-4.702)	0.541		
Docetaxel RECIST (SD-PD versus CR-PR)	9.154 (2.237-37.451)	0.002	8.608 (1.325-55.928)	0.024
Total RECIST (SD-PD versus CR-PR)	0.447 (0.167-1.199)	0.110		
pCR versus non-pCR	0.096 (0.011-0.812)	0.031	0.426 (0.037-4.907)	0.494
Miller-Payne stage				
G3 versus G1+G2	0.556 (0.147-2.103)	0.387		
G4+G5 versus G1+G2	0.111 (0.020-0.607)	0.011		
RFS(Recurrence-Free versus Recurrence)	8.455 (1.931-37.016)	0.005	7.356 (1.055-51.316)	0.044

## 4 Discussion

Taxanes, such as docetaxel, are cornerstone drugs in neoadjuvant chemotherapy for breast cancer. However, their efficacy is often curtailed by drug resistance, which is significantly associated with tumor aggressive phenotypes (19). This poses a substantial challenge in predicting and screening personalized treatments to enhance the pathological complete response rate (pCR) of breast cancer from standard chemotherapy regimens. Various models, including patient-derived tumor xenografts (PDX), patient-derived organoids (PDTO), and patient-derived tumor-like cell clusters (PTC), have been utilized to study tumor drug resistance mechanisms. Nevertheless, patient-derived primary cell lines (PCCLs) are gaining prominence as an ideal tool for predicting chemosensitivity due to their ability to retain the heterogeneity and microenvironment characteristics of the original tumor (13, 20–23). In this study, we successfully cultured four PCCLs, which could be passaged multiple times. Immunostaining confirmed that the phenotypic characteristics of these PCCLs are highly consistent with tumor tissues. Further CCK-8 experiments revealed a high consistency between the sensitivity of primary cell lines to docetaxel and the sensitivity of tumors to docetaxel in patients' neoadjuvant therapy. This finding indicates that PCCL technology could be employed to screen and predict the sensitivity of breast cancer to docetaxel chemotherapy, offering a promising tool for personalized treatment.

RNA sequencing and protein-protein interaction (PPI) network analysis of primary breast cancer cell lines, combined with the TCGA database, GEPIA2 survival-related genes, and GeneCards taxane (docetaxel) resistance-related genes, revealed a significant overexpression of the COL1A2 gene in docetaxel-resistant cell lines and its association with a poor prognosis in breast cancer patients. qRT-PCR validation has confirmed high COL1A2 expression in resistant cell lines and low expression in sensitive ones. Clinical studies demonstrated further that there is a negative correlation between COL1A2 expression and the pCR rate of neoadjuvant therapy, with patients exhibiting low COL1A2 expression being more likely to achieve pCR after docetaxel treatment. Furthermore, COL1A2 expression showed a significant correlation with patients' recurrence-free survival (RFS), a finding consistent with *in vitro* experiments.

Collagen type I (COL1) consists of two α1 chains (COL1A1) and one α2 chain (COL1A2) and is widely present in the extracellular matrix of bones, skin, and tendons. COL1A2 encodes the α2 chain of type I collagen and is overexpressed in various tumors, including gastric (16, 17), pancreatic (24), and colorectal cancers (25). It promotes tumor progression by enhancing cancer cell growth, invasion, metastasis, and drug resistance. Additionally, it is closely linked to poor cancer prognosis (15, 26–29). For instance, in lung adenocarcinoma, COL1A2 is associated with the cancer-associated fibroblast (CAF) subgroup, while in gastric cancer, COL1A2 overexpression increases cell resistance to apatinib (30, 31). Our qRT-PCR and immunohistochemistry analyses have shown significantly higher COL1A2 expression in docetaxel-resistant breast cancer cell lines and clinical samples compared to sensitive groups, with its cytoplasmic and extracellular matrix expression showing a negative correlation with neoadjuvant chemotherapy efficacy. Univariate and multivariate logistic regression analyses further indicated that high COL1A2 expression is significantly associated with poor response rates and reduced RFS in docetaxel treatment, suggesting its potential as a marker for predicting chemotherapy resistance in breast cancer.

Previous studies have generally associated high COL1A2 expression with tumor cell chemoresistance. Our immunohistochemistry analysis revealed that, in pathological sections of patients with poor neoadjuvant therapy response, tumor cells with high COL1A2 expression were often surrounded by collagen deposition zones, which correlated with a weak response to immunotherapy. This is likely related to type I collagen deposition, which alters the mechanical properties of the tumor microenvironment by remodeling the extracellular matrix, increasing its stiffness and rigidity (32). This process activates the integrin signaling pathway, inhibits chemotherapeutic drug penetration, and reduces the drug concentration within the tumor (33–35). Additionally, COL1A2 can directly bind to certain chemotherapeutic drugs, limiting drug-tumor cell contact and reducing drug efficacy (36). Other studies suggest COL1A2 activates the PI3K-Akt signaling pathway, enhancing tumor cell survival by binding to receptors like integrin α1β1 and α2β1 (28, 33, 37). Our results align with these viewpoints, which further confirm the correlation between high COL1A2 expression and neoadjuvant chemotherapy resistance, providing new insights into the relationship between breast cancer resistance mechanisms and the tumor microenvironment.

Studies on COL1A2 in breast cancer chemoresistance remain limited, particularly regarding the role of PCCL technology in verifying COL1A2's involvement in resistance. Our study found that COL1A2 was significantly overexpressed in docetaxel-resistant breast cancer cells via PCCL technology and was closely associated with patient docetaxel insensitivity, low neoadjuvant therapy pCR, and shorter RFS. Further experiments showed that high COL1A2 expression was closely linked to docetaxel resistance in luminal B HER2-negative breast cancer patients, providing an experimental basis for COL1A2 as a potential breast cancer chemoresistance marker.

In conclusion, our study suggests that PCCL technology can effectively predict breast cancer chemosensitivity, offering a valuable tool for personalized treatment. COL1A2 overexpression was found to be associated with docetaxel resistance and poor prognosis in breast cancer patients, suggesting its potential as a chemoresistance marker. Future research should explore further the mechanisms underlying COL1A2-mediated chemoresistance to develop targeted therapeutic strategies and improve treatment outcomes in breast cancer patients.

## 5 Conclusion

This study successfully established a primary breast cancer cell line, which retains the structural and functional characteristics of the original tumor. It was experimentally verified that this line can be used to predict the chemotherapy response of individual patients, providing a powerful tool for personalized chemotherapy drug selection. Sequencing data analysis of primary cell lines revealed a correlation between COL1A2 expression and docetaxel chemotherapy resistance. Further analysis of clinical pathological data confirmed that high COL1A2 expression was closely related to docetaxel resistance and poor patient prognosis, suggesting that it may serve as a potential marker for predicting docetaxel resistance in breast cancer.

However, this study has some limitations. Currently, only the correlation between COL1A2 expression and docetaxel resistance has been clarified, while the molecular mechanism remains unclear. Future studies should verify this correlation through additional basic experiments and a larger dataset of clinical samples. Moreover, an in-depth exploration of the interaction between COL1A2 and the tumor microenvironment is necessary to fully reveal its mechanism of action in breast cancer resistance.

## Data Availability

The data presented in this study are available from the NCBI Gene Expression Omnibus (GEO) under accession number GSE319840.

## Supplementary material

Supplementary Table 1 RNA-seq per-sample QC. Definitions. Read-level metrics (Total/Unique/Multi mapped; Mapping%) were computed from the alignment summary after merging lanes. Mapping% = Σ mapped reads / Σ total reads × 100. Unique% and Multi-mapping% were calculated as Σ uniquely mapped reads or Σ multi-mapped reads divided by Σ total reads × 100. Base-level metrics (Exon/Intron/Intergenic; Exonic rate) were derived from the regional assignment reports. Exonic rate (base-level) = Σ exonic bases / (Σ exonic + Σ intronic + Σ intergenic bases) × 100. Read-level and base-level metrics use different denominators (reads vs bases) and should not be cross-compared. QC aggregation. Q30 and GC are clean-read–weighted means aggregated across lanes (weights = clean reads per lane). Sanity checks. (Unique% + Multi-mapping%) should approximate Mapping% within ~3% at the read level; at the base level, totals are reported as Σ exonic + Σ intronic + Σ intergenic bases (no cross-level conservation is expected).
